# Intradermal Vaccination Induces Protective Immunity Against Foot-and-Mouth Disease While Mitigating Milk Yield Reduction in Ruminants

**DOI:** 10.3390/vaccines14080699

**Published:** 2026-08-13

**Authors:** Dong-Wan Kim, Seo-Yong Lee, So Eon Kim, Tae-Jun Kim, Hyejin Kim, Ji-Hyeon Hwang, Sun Young Park, Young-Joon Ko, Yoon-Hee Lee, Jong-Hyeon Park, Sung-Han Park

**Affiliations:** Center for FMD Vaccine Research, Animal and Plant Quarantine Agency, 177, Hyeoksin 8-ro, Gimcheon-si 39660, Gyeongsangbuk-do, Republic of Korea; dongwan12@korea.kr (D.-W.K.); happyhusband0406@korea.kr (S.-Y.L.); kse2384@korea.kr (S.E.K.); teajun0526@korea.kr (T.-J.K.); hyejin86@korea.kr (H.K.); jihyeonh87@korea.kr (J.-H.H.); sun3730@korea.kr (S.Y.P.); koyoungjoon@korea.kr (Y.-J.K.); lyhee74@korea.kr (Y.-H.L.); parkjhvet@korea.kr (J.-H.P.)

**Keywords:** foot-and-mouth disease, intradermal vaccination, ruminants, milk yield

## Abstract

Background: Foot-and-mouth disease (FMD) is a highly contagious viral disease causing substantial economic losses in livestock. Conventional intramuscular (IM) vaccination is widely used for FMD control but may be associated with transient reductions in milk production. This study evaluated whether intradermal (ID) vaccination could induce comparable immune responses and protection while minimizing potential impacts on productivity. Methods: In cattle, the immunogenicity of an experimental bivalent ID vaccine was compared with that of a commercially available IM vaccine by measuring antibody responses for up to 140 days post-vaccination (dpv). Milk production was monitored for 7 dpv in a separate cohort of lactating cattle. Protective efficacy was evaluated in goats following ID vaccination and challenge with a 2025 Republic of Korea serotype O field isolate (O/ME-SA/Ind-2001). Results: ID vaccination induced humoral immune responses comparable to those elicited by IM vaccination throughout the 140-day observation period. Although no statistically significant difference in milk production was observed, the ID group showed a smaller numerical reduction in milk yield. Following viral challenge, ID-vaccinated goats developed rapid neutralizing antibody responses, exhibited no clinical signs, and showed lower viral RNA levels than unvaccinated controls. Conclusions: Intradermal vaccination induced long-lasting immune responses comparable to conventional IM vaccination, was associated with a smaller numerical reduction in milk yield, and protected goats against challenge with a contemporary FMDV field isolate. These findings provide preliminary evidence supporting the potential of ID vaccination as an alternative strategy for FMD control in ruminants and warrant further comparative studies with conventional IM vaccination.

## 1. Introduction

Foot-and-mouth disease (FMD) is a highly contagious viral disease affecting cloven-hoofed animals and remains one of the most economically important livestock diseases worldwide. FMD outbreaks result in substantial economic losses due to reduced productivity, restrictions on animal movement and international trade, and the implementation of costly control measures [1,2]. Vaccination with inactivated vaccines remains the primary strategy for FMD prevention and control in endemic countries [2].

Conventional intramuscular (IM) vaccination is the standard route for FMD vaccination and provides effective protection against clinical disease. However, transient local injection-site reactions and short-term changes in milk production have been reported following vaccination in dairy cattle, although these effects are generally mild and inconsistent among studies [3,4,5]. Therefore, there is growing interest in alternative vaccination strategies that can maintain protective immunity while minimizing vaccination-associated adverse effects.

Intradermal (ID) vaccination has attracted increasing attention because the dermis contains abundant antigen-presenting cells (APCs), including dendritic cells and Langerhans cells, which efficiently initiate adaptive immune responses [6,7,8]. Consequently, ID vaccination has been investigated as an alternative vaccine delivery strategy to improve immune responses while minimizing tissue injury associated with intramuscular injection [6,9,10,11]. However, immune responses to FMD vaccines may differ among livestock species because of differences in skin structure, immune characteristics, antigen concentration, and adjuvant formulation. Therefore, findings obtained in swine cannot be directly extrapolated to ruminants, highlighting the need for species-specific evaluation of ID FMD vaccination [2,9].

Our previous study demonstrated that ID vaccination induced protective immune responses and reduced local injection-site reactions in swine [12]. However, information regarding the long-term immunogenicity, production-related effects, and protective efficacy of ID vaccination in ruminants remains limited [9]. Therefore, the present study was designed with two complementary objectives. First, we compared the immunogenicity and production-related effects of ID and conventional IM vaccination in cattle by evaluating long-term antibody responses and changes in milk production following vaccination. Second, we evaluated the protective efficacy of the experimental ID vaccine against a contemporary Korean serotype O FMDV field isolate in goats. These findings provide practical information regarding the potential application of ID vaccination as an alternative vaccination strategy for FMD control in ruminants.

## 2. Materials and Methods

### 2.1. Vaccine

The ID FMD vaccine was formulated as a bivalent inactivated vaccine containing FMD O/Boeun/SKR/2017 (O BE; GenBank accession number MG983730.1) and A/Yeoncheon/SKR/2017 (A YC; GenBank accession number KY766148.1) strains. The vaccine contained 20 μg of purified inactivated O/Boeun/SKR/2017 antigen and 15 μg of purified inactivated A/Yeoncheon/SKR/2017 antigen per dose. These antigens were formulated with 10% alum (ALHYDROGEL Aluminium Hydroxide Gel Adjuvant; InvivoGen, San Diego, CA, USA), 1% saponin (Sigma-Aldrich, St. Louis, MO, USA), and 15% Emulsigen-D (MVP Technologies, Omaha, NE, USA) as adjuvants [13]. A commercially available inactivated bivalent FMD vaccine (Boehringer Ingelheim, Bracknell, UK) was used for IM vaccination. The vaccine contained O1/Manisa, O/3039 (serotype O), and A22/Iraq (serotype A) antigens and had a reported potency of >6 PD50.

### 2.2. Vaccination in Cattle

The cattle experiment was designed to compare the immunogenicity and production-related effects of intradermal (ID) and conventional intramuscular (IM) vaccination.

The ID vaccine (0.5 mL/dose) was administered intradermally into the shoulder region using a Pulse 250 needle-free injection system (Pulse Needle-Free Systems, Lenexa, KS, USA), whereas the commercial vaccine (2.0 mL/dose) was administered intramuscularly into the shoulder region using a conventional needle and syringe.

To compare immunogenicity between vaccination routes, forty clinically healthy 8–10-week-old Holstein-Friesian calves from a field farm were randomly assigned to two groups (n = 20 per group) (Table 1).

A separate set of animals was used to evaluate the changes in milk yield following vaccination. Forty clinically healthy lactating female Holstein-Friesian cows from the same dairy farm were randomly assigned to the ID and IM groups (n = 20 per group). Cows were milked twice daily (morning and afternoon). Milk production was monitored for 7 dpv, and the rate of change in milk yield was compared between groups (Table 2).

Throughout the study, all cattle were maintained under identical feeding and management conditions, with feed provided according to the routine farm schedule.

### 2.3. Viral Challenge in Goats

The goat experiment was conducted independently to evaluate the protective efficacy of the experimental ID vaccine against a contemporary Korean FMDV field isolate. All animal procedures were approved by the Institutional Animal Care and Use Committee of the Animal and Plant Quarantine Agency (APQA), Republic of Korea (approval number: 2025–1920). Animals were housed in an ABSL-3 facility at the APQA and provided with feed and water ad libitum. For the challenge study, healthy 8-week-old goats were used. Eight seronegative animals identified by serological screening were randomly assigned to ID vaccination (n = 5) and unvaccinated control (n = 3) groups (Table 3). Animals were challenged with a serotype O FMD virus belonging to the O/ME-SA/Ind-2001 lineage, isolated during the 2025 outbreak in the Republic of Korea. Clinical signs were evaluated daily using a seven-point scoring system based on the distribution of FMD-associated lesions. One point was assigned for lesions detected on each hoof (maximum of four points), and one point each was assigned for lesions on the tongue, oral cavity, and nose, resulting in a maximum clinical score of seven. Clinical scoring was performed according to previously published criteria [14]. Viral RNA levels were assessed in nasal swab and serum samples.

### 2.4. Serological Analysis

To compare immunogenicity between vaccination routes, serum samples from cattle were collected at 0, 28, 56, 84, 112, and 140 days post-vaccination (dpv). For the challenge study, goat sera were collected at 0, 7, and 21 dpv and at 0, 1, 3, 5, and 7 days post-challenge (dpc). Serum samples were heat-inactivated at 56 °C for 1 h. Structural protein (SP) ELISA was performed to evaluate the vaccine-induced humoral antibody response, whereas the virus neutralization test (VNT) was used to assess functional neutralizing antibody responses against the homologous vaccine strains and the challenge virus. Type O ELISA was performed using a VDPro FMDV Type O AB ELISA kit (MEDIAN Diagnostics, Chuncheon, Republic of Korea). According to the manufacturer’s instructions, samples with an optical density (OD) value ≤ 0.6 were considered positive. For ease of data presentation, ELISA results are shown as OD ratio values calculated as (1-OD), such that OD ratio values ≥ 0.4 correspond to positive samples. Virus neutralization (VN) titers against the homologous type O and A vaccine strains and the challenge virus were determined according to the WOAH Terrestrial Manual [2].

### 2.5. Real-Time Reverse Transcription Polymerase Chain Reaction

Viral RNA was extracted from serum and nasal samples using a Maxwell RSC RNA extraction kit (Promega Corporation, Madison, WI, USA). FMDV RNA was detected using a One-Step PrimeScript RT–PCR Kit (Bioneer, Daejeon, Republic of Korea) and analyzed on a CFX96 Touch Real-Time PCR Detection System (Bio-Rad Laboratories, Hercules, CA, USA).

### 2.6. Statistical Analysis

Data are presented as mean ± standard error. Statistical significance was evaluated using two-way analysis of variance followed by Tukey’s multiple comparison test in Prism v.9 (GraphPad Software, San Diego, CA, USA). Statistical significance was set at * *p* < 0.05, ** *p* < 0.01, *** *p* < 0.001, and **** *p* < 0.0001; ns stands for not significant.

## 3. Results

### 3.1. Comparison of Immunogenicity Between ID and IM Vaccination in Cattle

**Figure 1:**
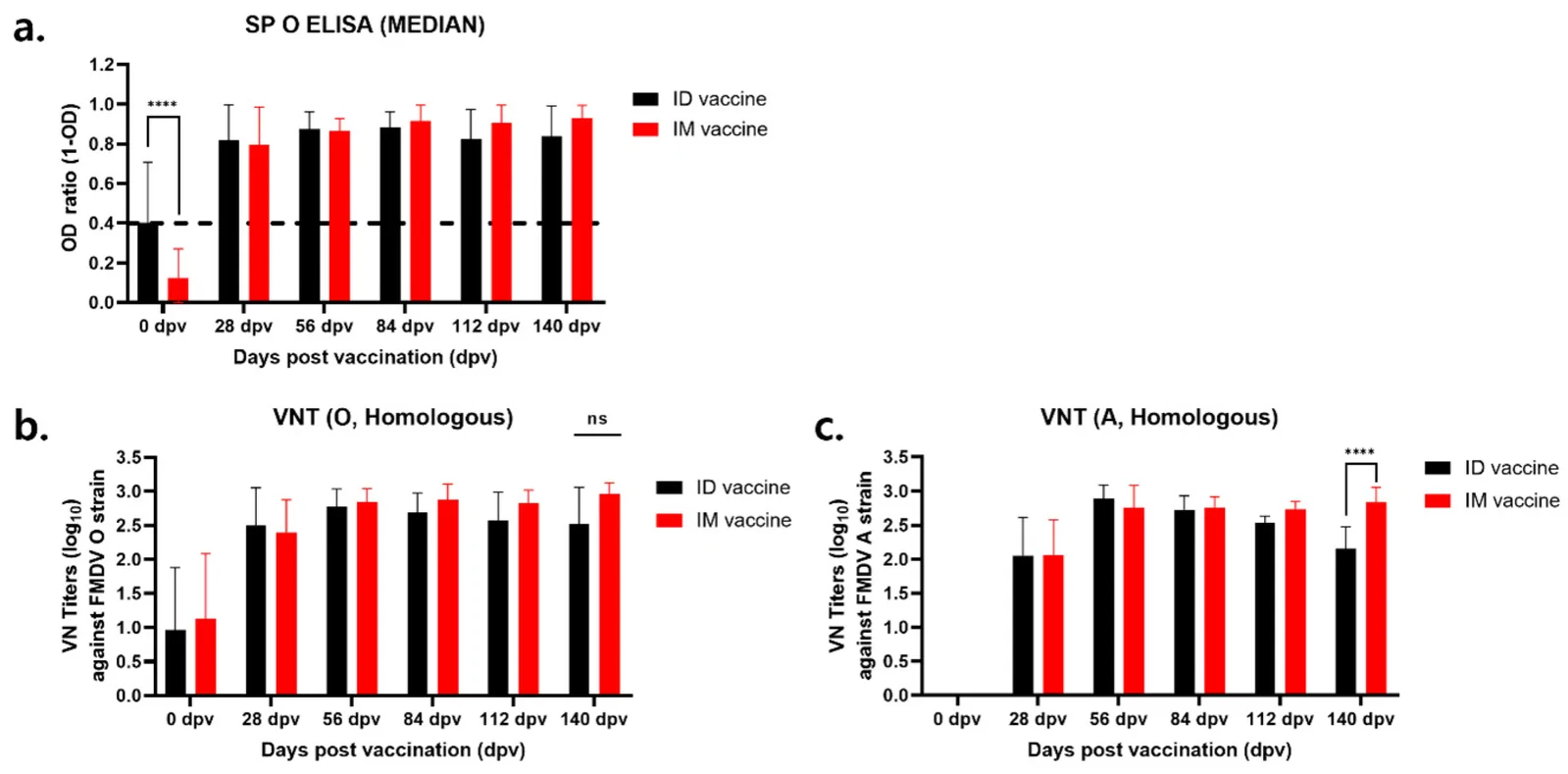
Immunogenicity of intradermal (ID) and intramuscular (IM) foot-and-mouth disease (FMD) vaccination in cattle. (**a**) Antibody titer measured by SP O ELISA. (**b**) Viral neutralization titers (VNT) against the homologous type O strain. (**c**) VNT against the homologous type A strain. SP, structural protein; FMDV, FMD virus; OD, optical density. **** *p* < 0.0001; ns, not significant. The black dotted line represents the positive cut-off threshold (OD ratio = 0.4).

Comparison of immunogenicity between the ID and IM groups showed that both groups developed antibody titers above the positive threshold by 28 dpv, which were maintained throughout the study period (Figure 1a).

Neutralizing antibody titers against the homologous type O strain reached 1:512 at 28 dpv and remained high thereafter, with no significant differences between groups (Figure 1b). Both groups maintained mean neutralizing antibody titers above 1:170 for up to 140 dpv.

Comparable responses were also observed against the type A strain, with titers ≥ 1:100 at 28 dpv (Figure 1c). Although the ID group showed a slight decrease in neutralizing antibody titers by 140 dpv, titers remained above 1:100.

### 3.2. Changes in Milk Yield Following Vaccination in Cattle

**Figure 2:**
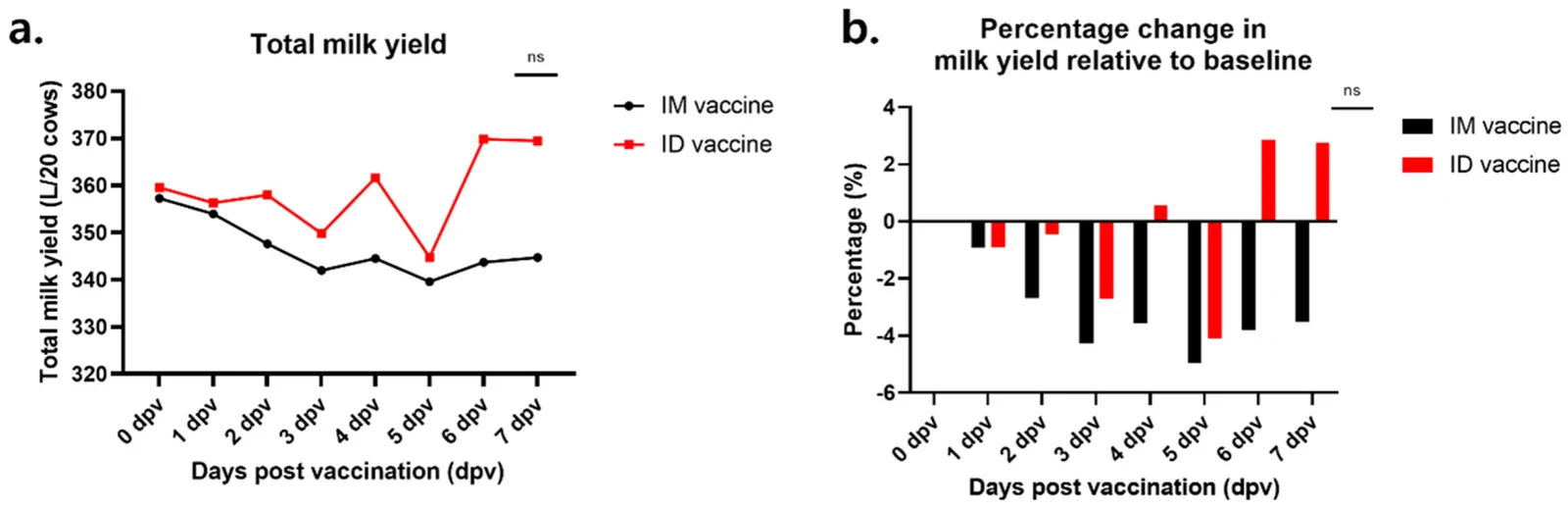
Comparison of changes in milk yield following intradermal (ID) and intramuscular (IM) vaccination in cattle. Percent changes in milk production were calculated relative to the total yield pre-vaccination. (**a**) Total daily milk yield of the ID and IM groups. (**b**) Change (%) in milk yield relative to baseline in each group. ns, not significant.

Milk yield was monitored for 7 dpv. Total daily milk yield in ID and IM groups was approximately 360 L (sum of 20 cattle) prior to vaccination. By 7 dpv, total milk yield decreased to 344 L in the IM group, whereas the ID group showed 369 L milk yield (Figure 2a). Both groups reached the lowest milk yield at 5 dpv; however, the ID group subsequently showed a recovery, returning to approximately baseline levels, whereas that of the IM group remained slightly reduced (Figure 2a). During the monitoring period, milk yield was reduced in 12 (60%) and 8 (40%) animals in the IM and ID groups, respectively.

No significant differences were observed between the groups; however, the IM group showed an average decrease of approximately 3% in milk yield, whereas the ID group showed a slight increase of approximately 2% relative to the baseline (Figure 2b). These findings suggest that ID vaccination was associated with a smaller numerical reduction in milk production.

### 3.3. Protective Efficacy of the ID Vaccine in Goats

**Figure 3:**
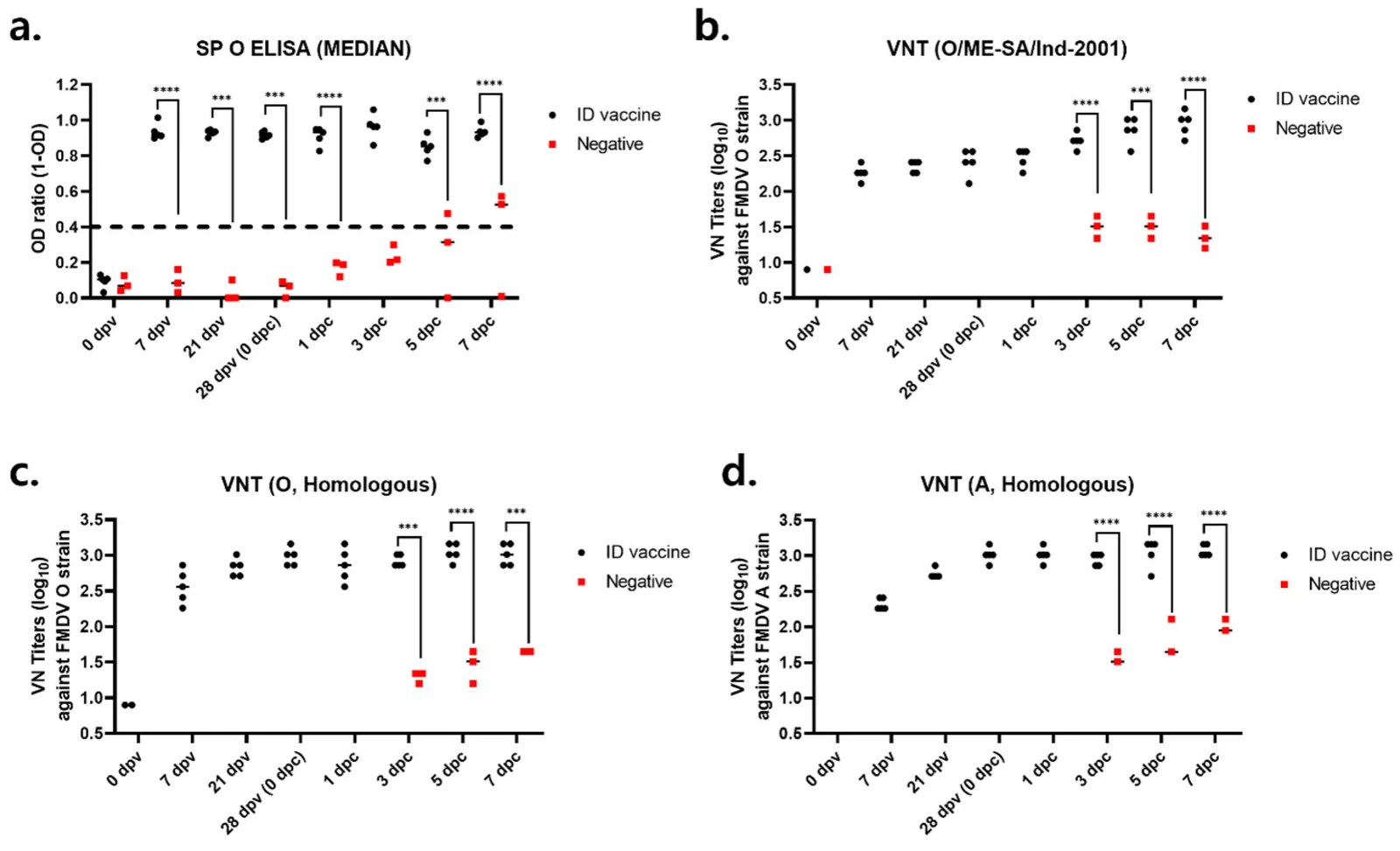
Immune responses in goats following challenge after intradermal (ID) vaccination. Neutralizing antibody titers are expressed as log_10_ values. (**a**) Antibody response measured by SP O ELISA. (**b**) Viral neutralization titers (VNT) against the challenge virus. (**c**) VNT against the homologous type O vaccine strain. (**d**) VNT against the homologous type A vaccine strain. SP, structural protein; dpc, days post-challenge; dpv, days post-vaccination; OD, optical density. *** *p* < 0.001; **** *p* < 0.0001.

Following ID vaccination, goats exhibited a rapid and robust immune response, with VN titers exceeding 1:100 as early as 7 dpv and reaching approximately 1:1000 by the end of the experiment (Figure 3b), with similar trends observed against homologous type O and A strains (Figure 3c,d).

**Figure 4:**
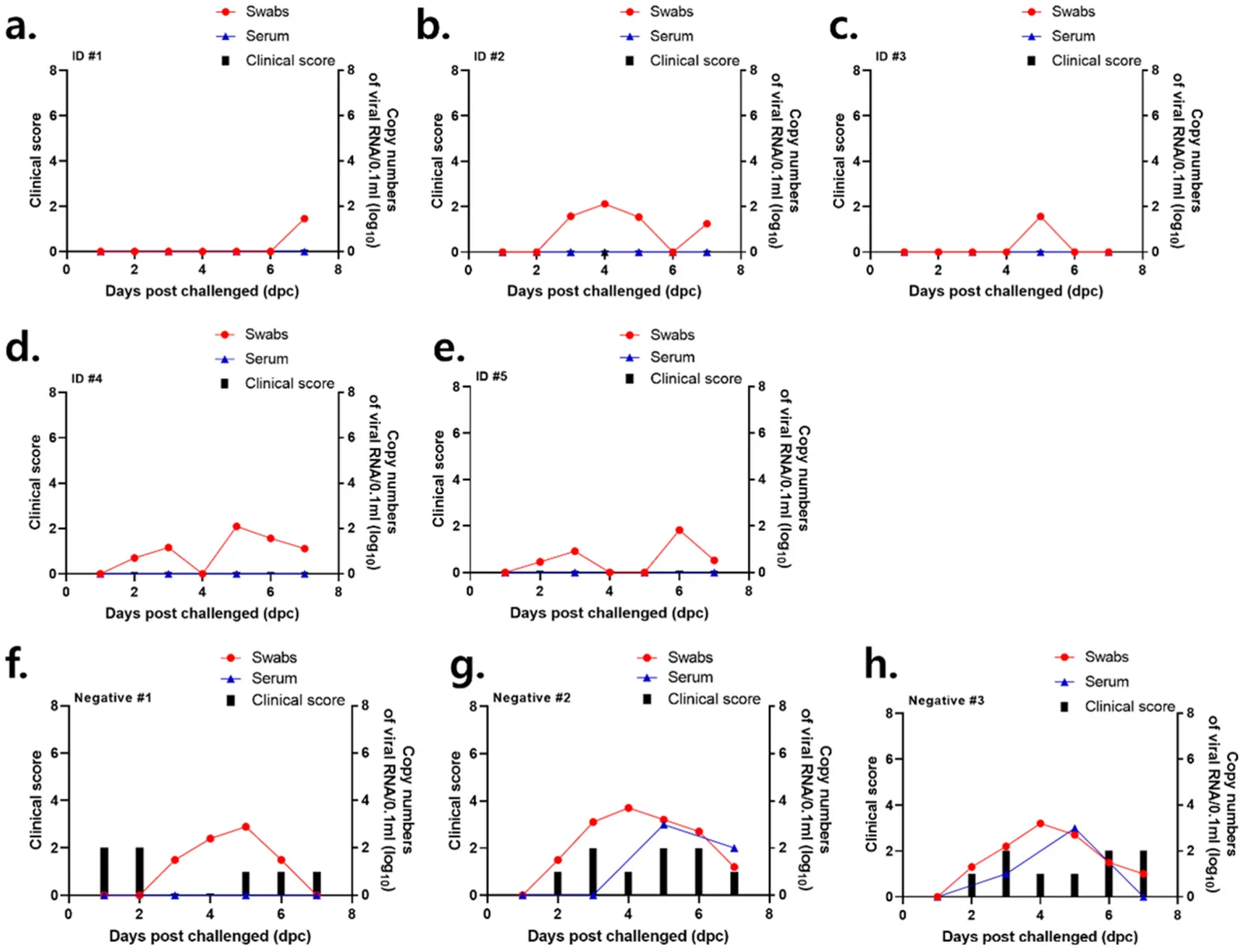
Clinical symptoms and viremia in intradermally vaccinated and control goats following challenge with foot-and-mouth disease (FMD) virus. (**a**–**e**) Viral RNA levels and clinical scores of individual intradermally vaccinated goats following challenge. (**f**–**h**) Viral RNA levels and clinical scores of individual unvaccinated control goats following challenge. ND indicates values below the limit of detection. No missing observations occurred during the challenge experiment. ID, intradermal; ND, not detected.

After challenge, none of the five ID-vaccinated goats developed clinical signs (clinical score = 0 throughout the observation period), whereas all three unvaccinated control goats developed clinical signs beginning at 1–2 dpc (Figure 4). Viral RNA levels in nasal swabs remained low in the ID-vaccinated group, with a maximum value of 2.122 (log10 copies), and no viremia was detected in serum (Figure 4a–e). In contrast, all unvaccinated control goats exhibited detectable viral RNA, with peak viremia occurring at 4–5 dpc and peak viral RNA levels ranging from 2.947 to 3.715 (log10 copies) (Figure 4f–h).

## 4. Discussion

This study demonstrated that intradermal (ID) vaccination induced humoral immune responses comparable to those elicited by conventional intramuscular (IM) vaccination in cattle. Although no statistically significant differences in milk production were observed between the two vaccination routes, the ID group showed a smaller numerical reduction in milk yield than the IM group during the observation period. These findings suggest that ID vaccination may represent a feasible alternative to conventional IM vaccination in cattle, providing comparable immunogenicity without evidence of a significant adverse effect on milk production.

In goats, ID vaccination elicited a rapid humoral immune response and provided protection against FMDV challenge, as demonstrated by the absence of clinical signs and lower viral RNA levels than those observed in unvaccinated controls. Previous studies have demonstrated the feasibility and protective efficacy of ID FMD vaccination in swine [12,13,15]. The present findings provide preliminary evidence that ID vaccination can induce protective immune responses in ruminants. Nevertheless, because immune responses to FMD vaccines may differ among livestock species, these findings should be interpreted as evidence supporting the applicability of ID vaccination in ruminants rather than as a direct extrapolation from previous swine studies.

The tendency toward a smaller reduction in milk production following ID vaccination may be related to the distinct tissue environment targeted by dermal antigen delivery. Unlike IM vaccination, which deposits vaccine into muscle tissue, ID vaccination delivers antigen to the dermis, where abundant antigen-presenting cells, including dendritic cells and Langerhans cells, facilitate efficient immune activation while potentially reducing local tissue injury [6,7,8]. Although local inflammatory responses, injection-site reactions (e.g., swelling and pain), systemic adverse reactions (e.g., fever), and device-related delivery performance were not systematically evaluated in the present study, reduced tissue injury following ID vaccination may have contributed to the observed trend in milk production. Further investigation incorporating histopathological examination and assessment of local inflammatory biomarkers would help clarify the biological mechanisms underlying this observation. In addition, milk yield was evaluated relative to a single pre-vaccination baseline, and potentially relevant cow-level variables such as parity, days in milk, and pregnancy status were not incorporated into the analysis. Therefore, the observed differences in milk yield should be interpreted as exploratory numerical trends rather than evidence of a definitive productivity benefit.

One limitation of the present study is that the goat challenge experiment included only an ID-vaccinated group and an unvaccinated control group. Therefore, although the study demonstrated the protective efficacy of the ID vaccine against FMDV challenge, it was not designed to directly compare the protective efficacy of ID and conventional IM vaccination. In addition, the commercial IM vaccine and the experimental ID vaccine were not identical formulations, and differences in antigen composition, adjuvant formulation, or manufacturing process may have influenced the observed responses. These limitations should be considered when interpreting the findings. Nevertheless, the present study provides preliminary evidence that ID vaccination induces comparable humoral immune responses in cattle and confers protection against FMDV challenge in goats, supporting further evaluation of this vaccination strategy in ruminants.

Evaluation of the early protective efficacy of ID vaccination in ruminants remains warranted. Previous studies in cattle have demonstrated that intradermal antigen delivery can induce early cytokine responses, including interferon gamma, interferon gamma-induced protein 10, and interleukin 6, suggesting activation of skin-resident immune cells [16]. Likewise, early protection following intradermal FMD vaccination has been demonstrated in swine [13]. Further investigation of early protective immunity together with local cytokine responses and cellular immune mechanisms would improve understanding of the immunological basis of ID vaccination in ruminants [17].

## 5. Conclusions

Intradermal vaccination induced humoral immune responses comparable to those elicited by conventional intramuscular vaccination in cattle and provided protection against FMDV challenge in goats relative to unvaccinated controls. Although no statistically significant differences in milk production were observed, ID vaccination was associated with a smaller numerical reduction in milk yield during the observation period. These findings provide preliminary evidence supporting the potential of intradermal vaccination as an alternative vaccination strategy for FMD control in ruminants and warrant further comparative studies with conventional IM vaccination.

## Figures and Tables

**Table 1:** Study design for comparison of immunogenicity between vaccination routes in cattle.

Group	No. of Cattle	Vaccine Strain	Serum Obtained at *dpv
ID	20	O BE + A YC	0, 28, 56, 84, 112, 140
IM (Commercial)	20	Commercial	

* dpv, day post-vaccination; ID, intradermal; IM, intramuscular; “O BE” and “A YC” refer to the FMDV vaccine strains O/SKR/BE/2017 and A/SKR/YC/2017, respectively.

**Table 2:** Study design for comparison of milk yield changes between vaccination routes in cattle.

Group	No. of Cattle	Vaccine Strain	Milk Yield Measurement Period (*dpv)
ID	20	O BE + A YC	0–7
IM (Commercial)	20	Commercial	

* dpv, day post-vaccination; ID, intradermal; IM, intramuscular.

**Table 3:** Study design for evaluation of protective efficacy of the ID vaccine in goats.

Group	No. of Goats	Vaccine Strain	Challenge Virus	Serum Obtained at dpc	Swab Obtained at dpc
ID	5	O BE + A YC	O/ME-SA/Ind-2001	−28, −21, −7, 0, 1, 3, 5, 7	1–7
Unvaccinated	3	-			

dpc, day post-challenge; ID, intradermal.

## Data Availability

The data supporting the findings of this study are available from the corresponding author upon reasonable request. They are not publicly accessible due to institutional policies.

## Abbreviations

The following abbreviations are used in this manuscript:

dpc	Day post-challenge
dpv	Day post-vaccination
ELISA	Enzyme-linked immunosorbent assay
FMDV	Foot-and-mouth disease virus
ID	Intradermal
IM	Intramuscular
SP	Structural protein
VN	Virus neutralization
VNT	Virus neutralization test
